# Laser guided ionic wind

**DOI:** 10.1038/s41598-018-31993-3

**Published:** 2018-09-10

**Authors:** Shengzhe Du, Tie-Jun Wang, Zhongbin Zhu, Yaoxiang Liu, Na Chen, Jianhao Zhang, Hao Guo, Haiyi Sun, Jingjing Ju, Cheng Wang, Jiansheng Liu, See Leang Chin, Ruxin Li, Zhizhan Xu

**Affiliations:** 1 State Key Laboratory of High Field Laser Physics, Shanghai Institute of Optics and Fine Mechanics, Chinese Academy of Sciences, Shanghai, China; 2 Centre d’Optique, Photonique et Laser (COPL) and Département de physique, de génie physique et d’optique, Université Laval, Québec, Québec, G1V 0A6 Canada

## Abstract

We report on a method to experimentally generate ionic wind by coupling an external large electric field with an intense femtosecond laser induced air plasma channel. The measured ionic wind velocity could be as strong as >4 m/s. It could be optimized by increasing the strength of the applied electric field and the volume of the laser induced plasma channel. The experimental observation was qualitatively confirmed by a numerical simulation of spatial distribution of the electric field. The ionic wind can be generated outside a high-voltage geometry, even at remote distances.

## Introduction

Ionic wind, also called corona wind or electric wind^1,2^, is an air flow generally driven by the electric field created by applying a high voltage to an electrode system. Initially the electrons in the ambient air resulting from ionization of air molecules by cosmic rays are accelerated under the large electric field near the electrode and undergo inelastic collisions. Consequently, more molecules are ionized in the process of electron avalanche ionization. The ions (positive ions in positive corona and negative ions, namely O_2_^−^, in negative corona) resulting from the ionization in air are also accelerated by the electric field away from the electrode and transfer their momenta to air particles via collisions, initiating a drag of the bulk air which is referred to as the ionic wind. Ionic wind has raised great interests and has found applications^3–9^. Due to their robustness, simplicity, low power consumption, and ability for real-time control at high frequency, plasma based actuators have been widely used in aerodynamic applications^3^. As a successful demonstration, Deep Space 1, the first mission of NASA’s New Millennium Program propelled by ion thruster engine, was launched on October 24, 1998^4,5^. An ionic wind generator has been suggested for a next-generation cooling device for LEDs and other electronic devices because of its high cooling performance, light and compact size, low noise, immune from vibration etc.^6^. The mobilities of ions from ionic wind created by corona discharges has also been used for gas diagnostics, for instance to detect and measure gas contaminants through the mobility spectrum of the ions^7^. In addition, ionic wind could induce precipitations such as rain and snow formation in a cloud chamber^8,9^. Traditionally, an ionic wind generator strictly relies upon the configuration design of electrodes, such as needle-to-plate and needle-to-cylinder types^3–9^. Those setups are mostly limited in different kinds of restricted space or fixed location due to the fixed mechanical design of the electrodes.

In this work we report on a brand new method to generate ionic wind by coupling a large electric field with an intense femtosecond laser ionized air plasma channel, namely filament. Large electric field was efficiently guided along the laser ionized air plasma channel resulting in the discharges at the end of the channel for ionic wind generation. This method is robust and immune to the specific design of traditional ionic wind generator, which also has potential application at a distance, even at remote distances.

## Experimental Setup

**Figure 1:**
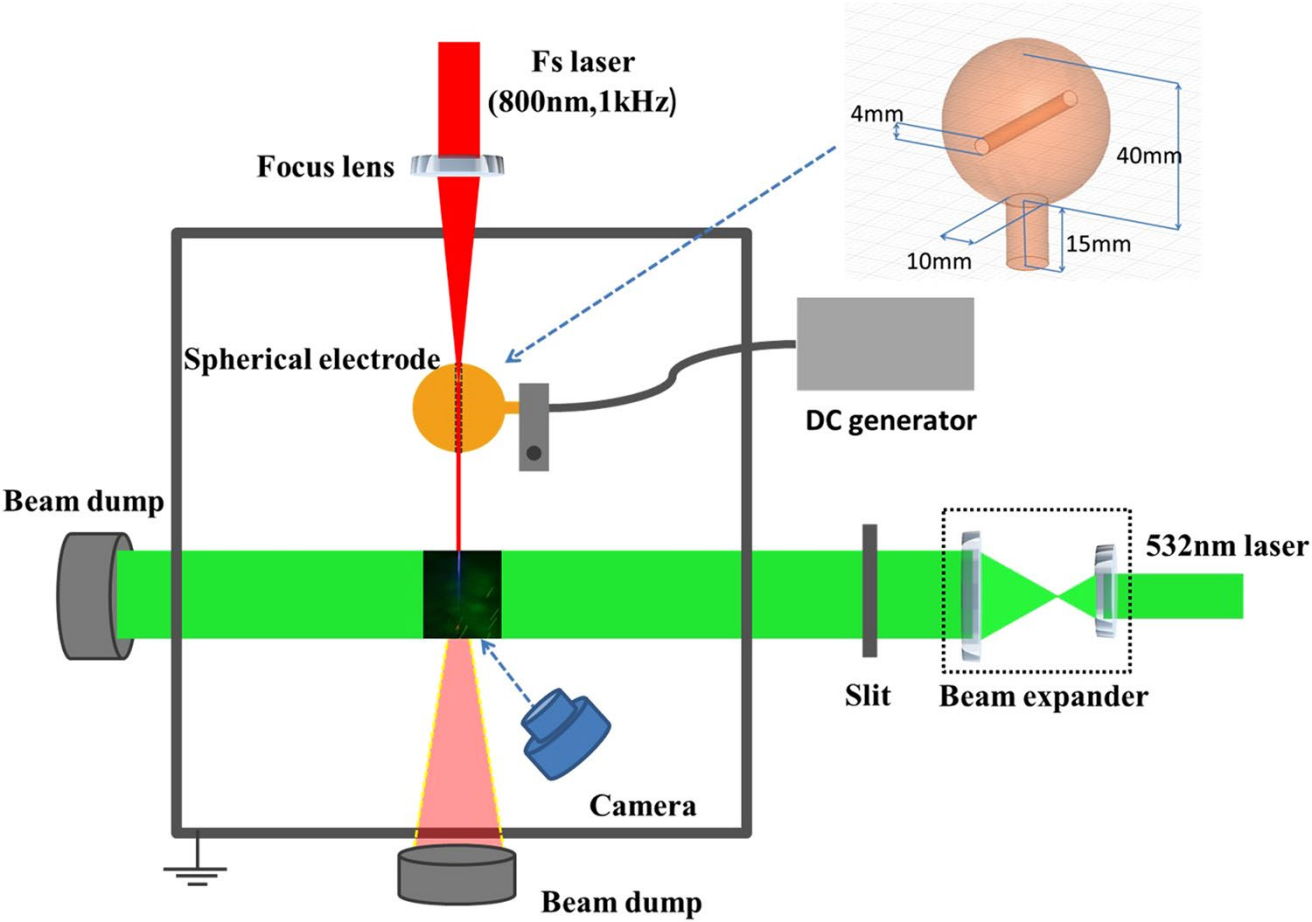
Experimental setup for ionic wind generation by laser guided discharges. The spherical electrode was held with an insulated plastic holder. The electrode design is shown in the inset. A good coupling was achieved by sending laser air plasma channel through the cylindrical channel which was punched through the spherical electrode.

A schematic of the experimental setup is shown in Fig. 1. Experiments were conducted using a Ti:sapphire chirped pulse amplification (CPA) laser system producing pulse energy of up to 6.85 mJ with central wavelength of 800 nm at a repetition of 1 kHz. The full-width half-maximum (FWHM) length of each pulse is 25 fs. The laser beam was focused by a convex lens with a focal length of 50 cm to create a stable plasma channel into a home-made Faraday cage. A spherical copper electrode with a special structure was designed to efficiently couple a large electric field to the plasma channel^10^. The purpose of using the specific electrode is two-fold: one is to try to avoid the corona from the electrode itself so that there is no wind blowing out from the electrode; the other is to increase the coupling efficiency between the electrode and the plasma channel so that more efficient laser guided discharges as well as stronger ionic wind could be expected. The main part of the electrode is a copper ball with a diameter of approximately 40 mm and there is a cylindrical channel punched through the ball as shown as the inset of Fig. 1. The diameter of the cylinder is 4 mm which is wide enough for the plasma channels to get through. The spherical copper electrode was connected to a DC high voltage power supply with a maximal output of positive 100 kV/1000 W.

Two methods were adapted to monitor the ionic wind velocity. One is based on the imaging of moving particles as shown in Fig. 1. Coarse particles (CaCO_3_/CaSO_4_ powders) were sprayed by hand into the Faraday cage. The diameter of these coarse particles is about 20–100 μm. A continuous wave (CW) 532-nm laser beam with 3.0 W output power after being expanded in diameter and truncated by a 30 mm (length) × 6 mm (width) slit was used to illuminate the particles. The slice of the laser beam passed through the horizontal plane where the 800 nm Ti: sapphire laser pulses propagated at 90 deg. as shown in Fig. 1. By recording the scattered 532 nm laser from moving particles with a digital camera of up to 60 frames per second (Nikon D7200), ionic wind velocity in different regions was estimated. Each particle with a velocity will fly for a distance during the camera exposure time, S. As a consequence, a trajectory of the particle’s movement is recorded in one frame for the exposure time. The velocity was calculated by simply doing division between the distance of particle trajectory and the exposure time. Note that the actual ionic wind velocity should be higher than the measured value, owing to a larger average mass of the coarse particles than the air molecules. The second method was based on a hot wire anemometer probe (HHF-SD1). The probe was placed 15 cm away from the ionization region around the tip of the plasma channels to directly measure the ionic wind in a relatively far and safe area.

## Results and Discussion

### Applied voltage dependence

**Figure 2:**
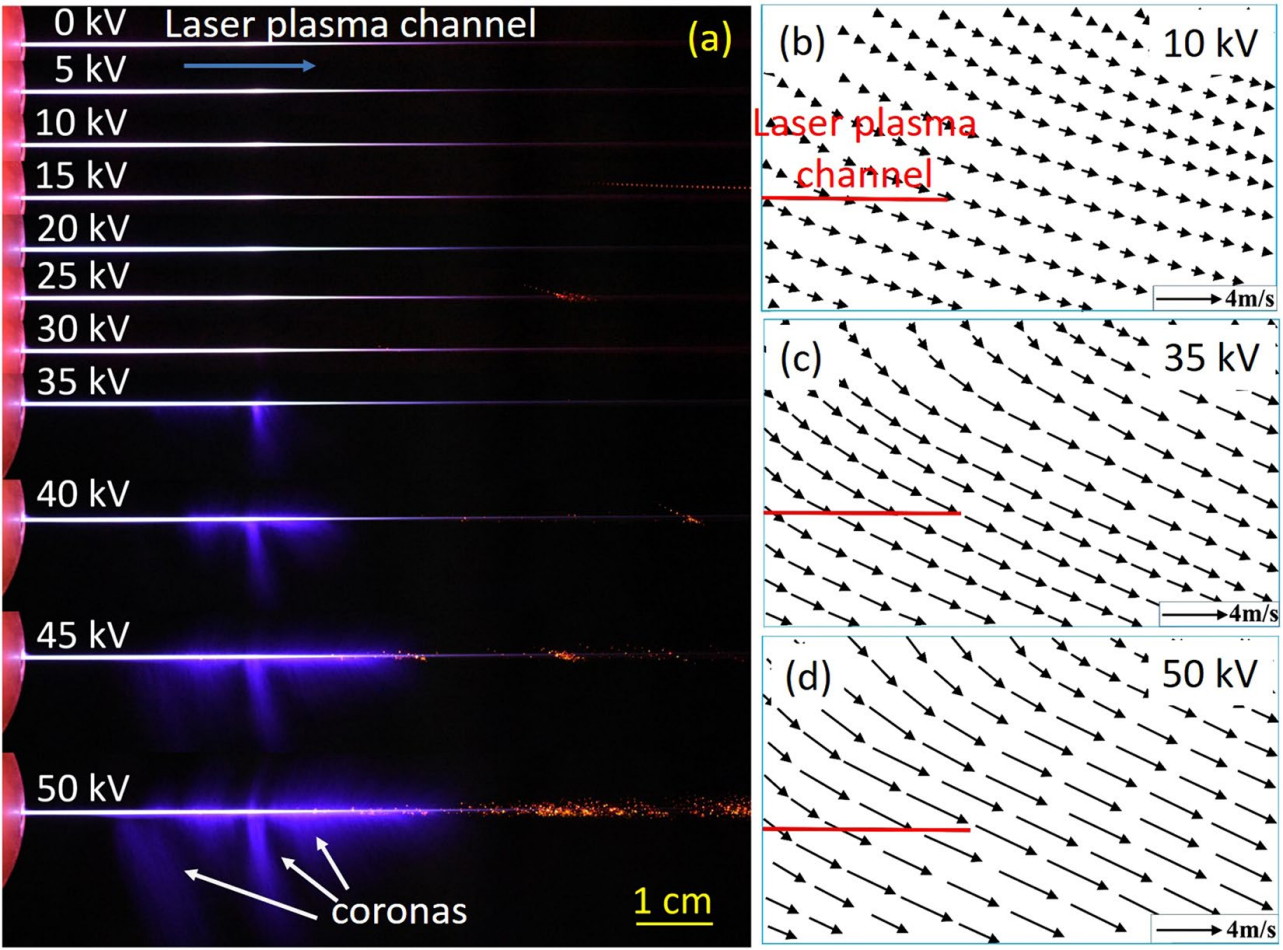
(**a**) Real-color images of discharges generated along the laser plasma channel in air with applied voltages ranging from 0 kV to 50 kV, and (**b**–**d**) the 2D flow fields of the ionic wind at the voltages of 10 kV, 35 kV, and 50 kV, respectively. Clear discharges guided by laser were assigned by the arrows. The recording parameters of the camera for (**b**–**d**) are S = 1/50 s, F = 4, and ISO = 25600 (**b**) S = 1/200 s, F = 4, and ISO = 25600 (**c**) S = 1/500 s, F = 4, and ISO = 25600 (**d**), respectively.

The plasma channel passed through the cylindrical hole of the electrode (Fig. 2(a)). The applied voltage was tuned from 0 kV to 50 kV. Leader and streamer types of corona discharges occurred around the tip and the length of the plasma channels (Fig. 2(a))^11^. At low applied voltage of <30 kV, only leader type of discharges along the laser plasma channel was observed. When the voltage was further increased (the voltage is >35 kV in Fig. 2(a)), streamer type of discharges along the laser plasma channel occurred and became much more significant. Note that the newly generated streamers of the discharges in Fig. 2(a) are not symmetrical along the plasma channel because of the asymmetry of the copper electrode. After spraying coarse particles into the cage, asymmetric particle trajectories around the plasma channel driven by ionic wind were clearly observed and recorded by the camera. A short video of the movement at the voltage of 20 kV can be seen in Supplementary Video S1. The 2D flows of the ionic wind obtained from particle movement images in the horizontal plane are shown in Fig. 2(b–d) for the voltages of 10 kV, 35 kV and 50 kV, respectively. The red lines in Fig. 2(b–d) are the plasma channel outside the electrode at zero volt. A clear asymmetry of the wind flows is seen.

**Figure 3:**
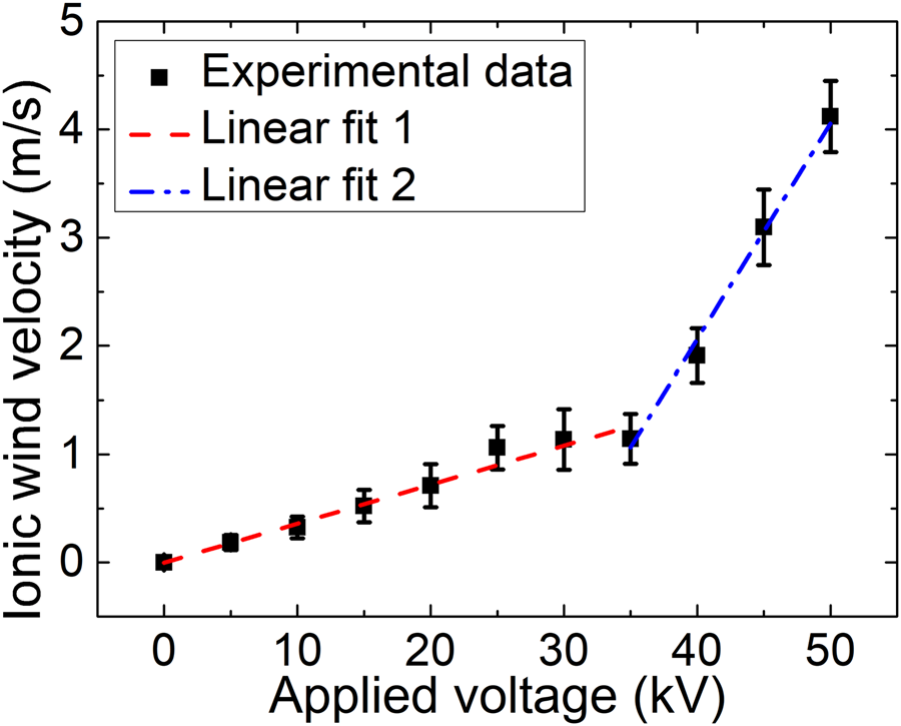
The maximum ionic wind velocity as a function of the applied voltage. Squared points are the measurement results and the red dashed and blue dashed dot line are the linear fits.

From the recorded images of particles’ movements, maximum velocities of these coarse particles were calculated under different high voltages (Fig. 3). The computed rectangular (9.99 cm × 6.65 cm) area in Fig. 2 was set near the front tip of the plasma channel where maximum velocities were observed. The background particles (applied voltage = 0 kV) slowly floated around with a velocity of ~0.05 m/s. When large electric field was coupled onto the air plasma channel, there were two regimes of maximum velocity of air flow when the applied voltage was increased as shown in Fig. 3: in the first regime from 0 kV to ~30 kV, the velocity was linearly proportional to the voltage; the velocity was also linearly proportional to the voltage in the second regime when the voltage was above 35 kV, but with a larger slope. This behavior is different from the trend of the ionic wind induced by a metallic electrode, in which only one linear dependence was reported^12^. A maximum velocity of >4 m/s was measured at the voltage of 50 kV.

### Air plasma length dependence

**Figure 4:**
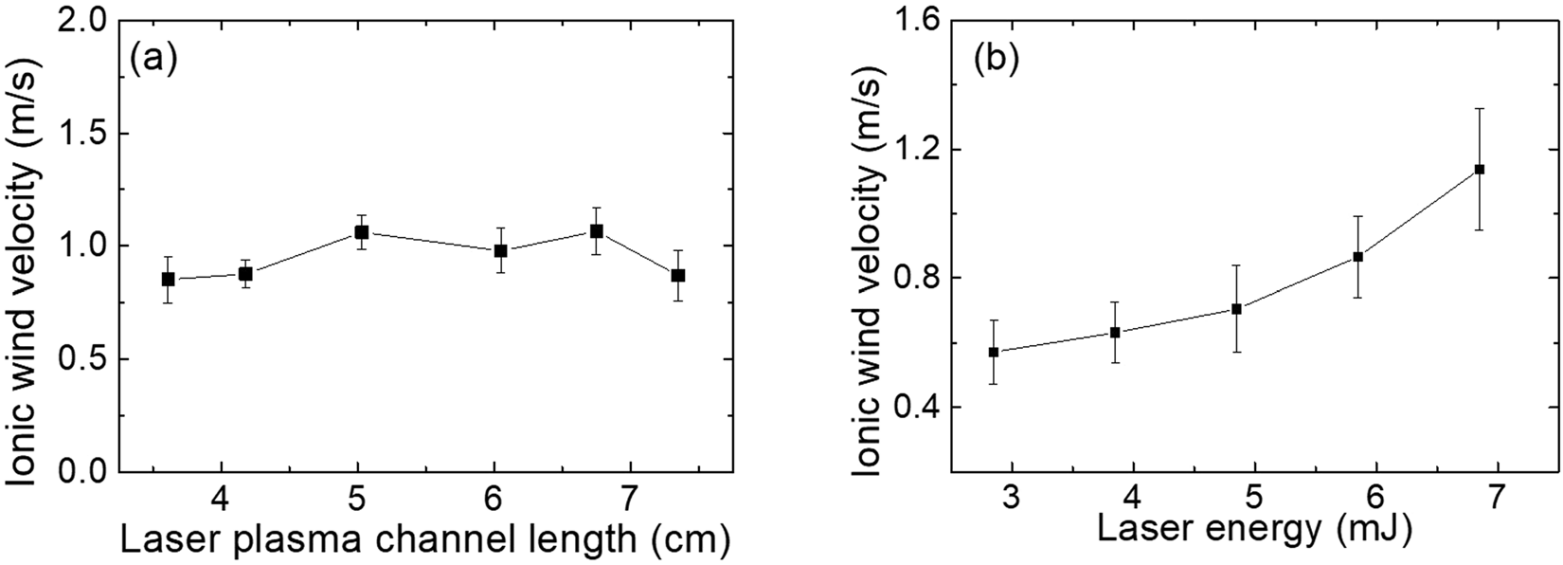
Measured ionic wind velocity as a function of (**a**) air plasma length outside the electrode and (**b**) the laser pulse energy. See more details in the text.

By keeping the laser pulse at 1 kHz/25 fs/6.85 mJ and the applied voltage at 30 kV where mostly leader type of discharges was observed at the plasma channel tip, the dependence of the ionic wind velocity on the air plasma length (which was defined as the length outside the electrode) was investigated by moving the laser plasma channel through the electrode. The evaluation area of wind speed was moved together with the plasma channel so that the area was always set near the front tip of the plasma channel where maximum velocities were observed. As shown in Fig. 4(a), when the external plasma length was tuned from 3.61 cm to 7.35 cm, the resultant wind velocity was almost constant. No significant change was observed. Since the length of the plasma channel could be controlled by altering a number of different laser parameters, such as energy, chirp, numerical aperture etc., from sub-meter to several tens of meters^13,14^, the air plasma guided ionic wind can be potentially generated at a distance from the electrode, even at remote distances.

In another experiment, the volume and the plasma density of the channel were changed by varying the laser pulse energy. The high voltage was fixed at 30 kV. When the laser energy was tuned from 2.85 mJ to 6.85 mJ, the maximum ionic wind velocity varied from 0.57 m/s up to 1.15 m/s (Fig. 4(b)). Two times increase in ionic wind was achieved. This result indicates that the air plasma channel induced ionic wind could be enhanced by improving the pulse energy. By increasing the laser pulse energy, a larger volume of plasma channel with higher electron density (lower impedance)^15^ may contribute the enhancement of ionic wind.

### Angular distribution of the ionic wind

**Figure 5:**
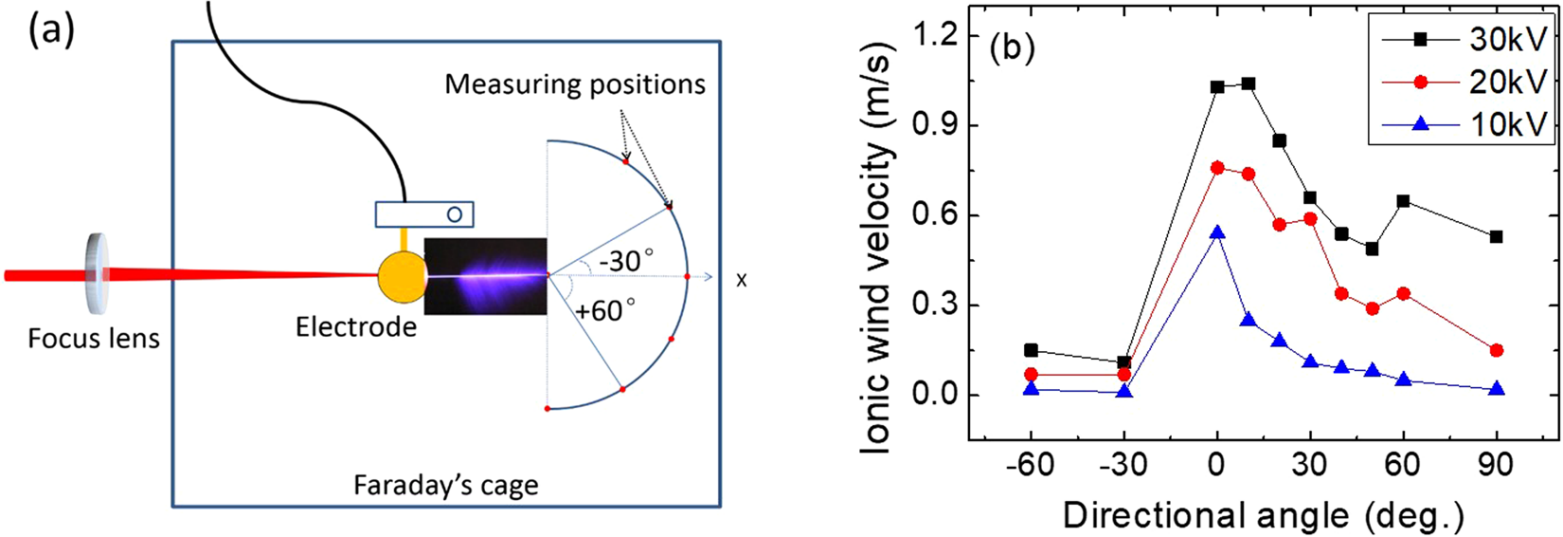
(**a**) The schematic of directional angle measurement of ionic wind. The electrode was held by an insulated plastic holder as shown in the figure. (**b**) The measured maximum ionic wind velocity as a function of detection angle. 1 kHz/25 fs/6.85 mJ femtosecond laser pulse was used to create plasma channel with a 50 cm focal length lens. The high voltage was fixed at 10 kV, 20 kV and 30 kV, respectively.

By using a hot wire anemometer probe (HHF-SD1), the ionic wind velocity was measured at a radial distance of 15 cm from the tip of the laser plasma channel. We note that the hot wire anemometer is a thin metallic wire based sensor, which cannot work at the corona region because of the strong electric field. The sensor has to be put at a distance away from the coronas so that it works accurately. The flow field further downstream could be measured which can provide the angular distribution information of the ionic wind. The detection schematic is shown in Fig. 5(a). Laser propagation direction is defined as angle of 0 degree. The angle in the clockwise direction is positive. 1 kHz/25 fs/6.85 mJ femtosecond laser pulses were used to create plasma channels with a 50 cm focal length lens. The high voltages were fixed at 10 kV, 20 kV and 30 kV, respectively. To reduce the influence of large electric field on the anemometer, an electrostatic shielding to the probe was done by twining grounded metallic mesh on the surface. As shown in Fig. 5(b), peak velocity occurs at 0 deg., which is the direction of laser propagation. Higher applied electric field strengths generate more discharges that, in turn, increase the flow speed. The flow field is asymmetrical because of the asymmetry of discharges which agree with the observation in Fig. 2(b–d).

### Numerical consideration and discussions

**Figure 6:**
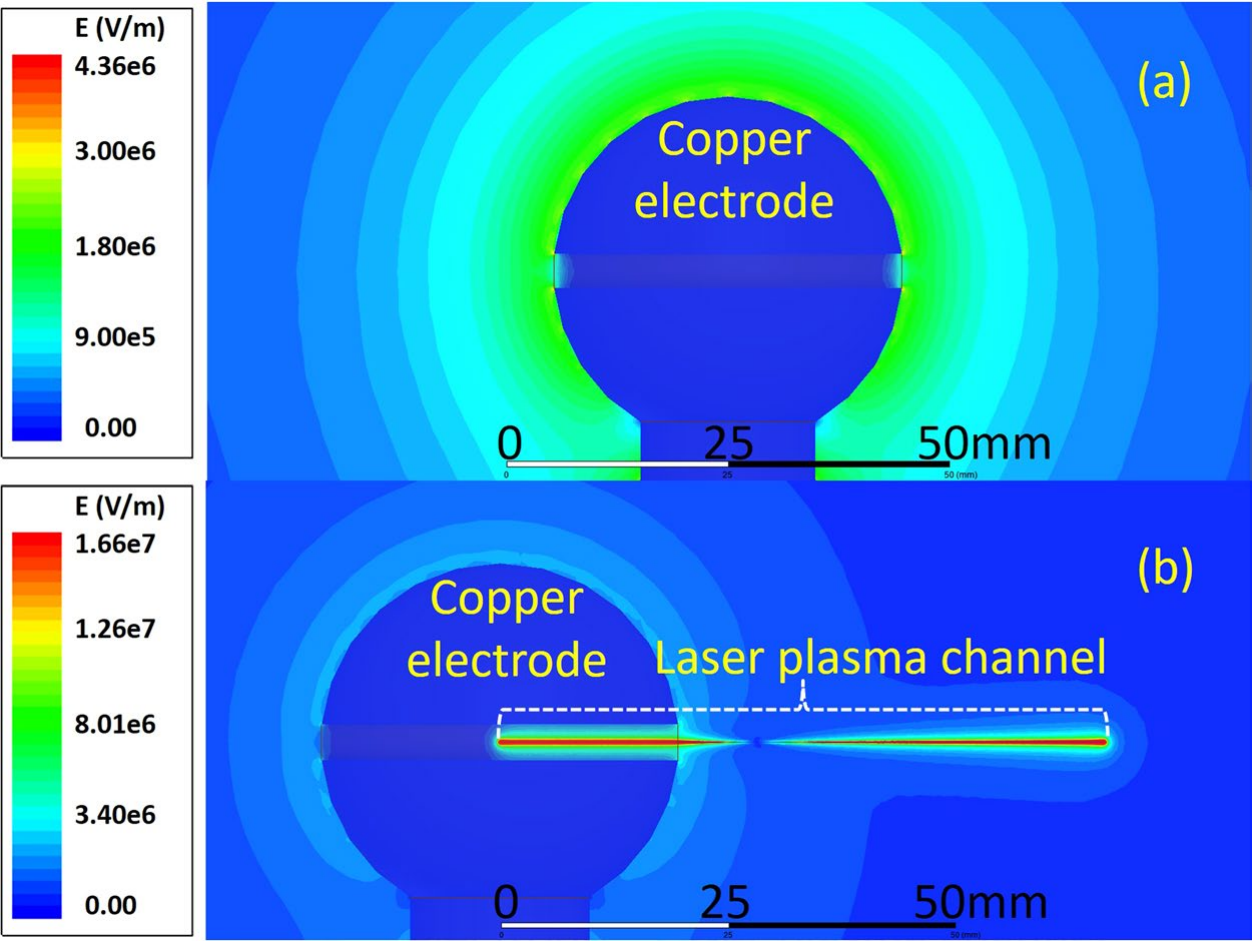
Electric field distribution around the spherical electrode when a high voltage of 50 kV was used (**a**) without laser plasma channel and (**b**) with laser plasma channel.

**Figure 7:**
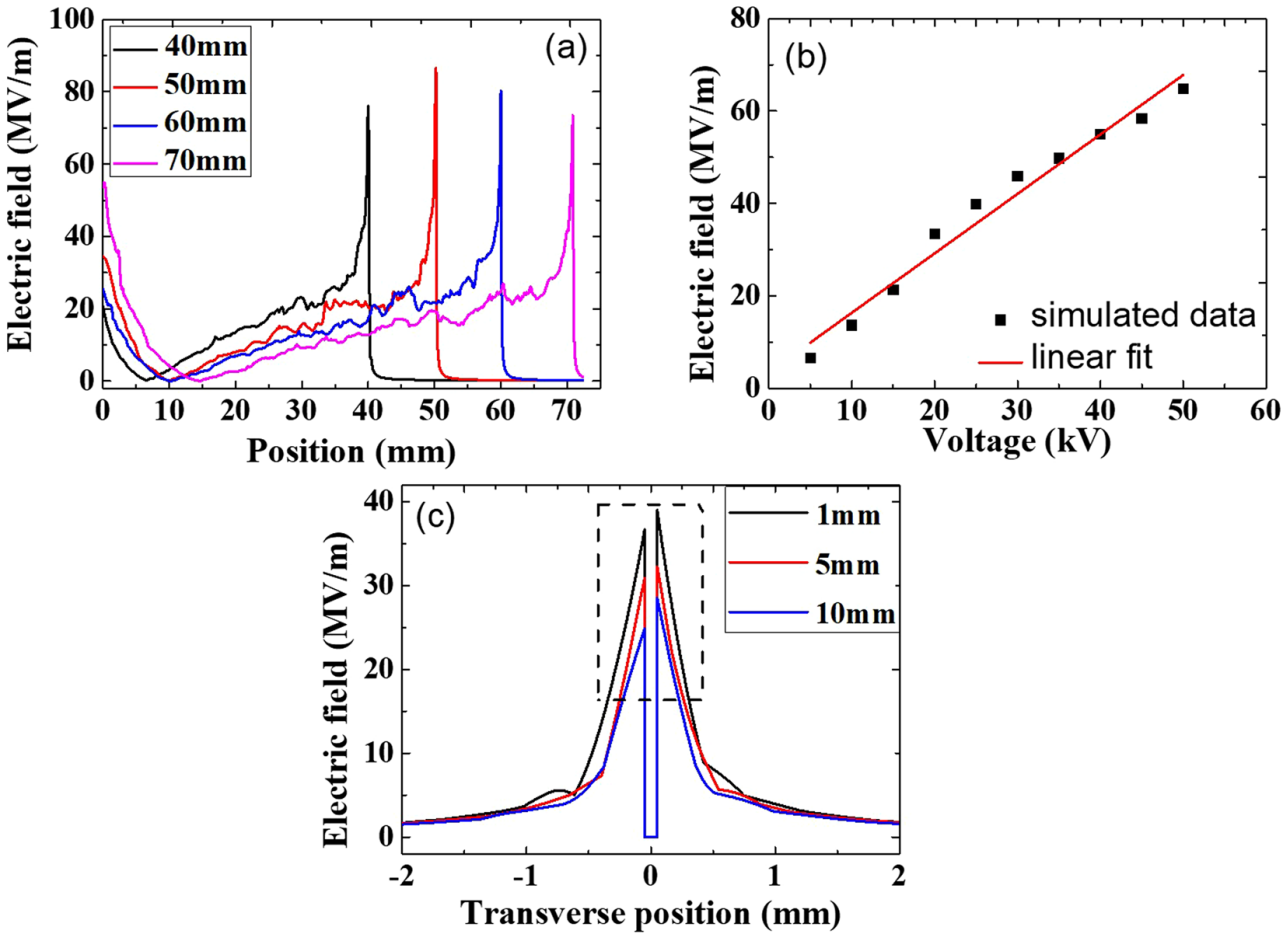
simulated electric field around the laser plasma channel end: (**a**) electric field distribution around the laser plasma channel end under different filament lengths of 40 mm, 50 mm, 60 mm, and 70 mm. The applied voltage was 50 kV; (**b**) the peak electric field around the end of 60 mm long plasma channel as a function of applied voltage. (**c**) the electric field distribution in the direction perpendicular to the laser propagation direction at three positions of 1 mm, 5 mm and 10 mm before the filament ends. The laser plasma channel position is defined as 0 and the negative transverse position is the side where the electrode was connected to the power supply (See Figs 1 and 5a).

In order to have a spatial distribution of the electric field **E** around the air plasma channel, the following Poisson equation and Maxwell equation were solved through finite element analysis:

1 $$\nabla \cdot ({{\rm{\varepsilon }}}_{{\rm{r}}}\cdot {{\rm{\varepsilon }}}_{0}\nabla {\rm{\Phi }})=-\,{{\rm{\rho }}}_{{\rm{V}}}$$

2 $${\rm{E}}=-\,\nabla {\rm{\Phi }}$$

where ε_r_ is the relative dielectric constant, ε_0_ the permittivity of vacuum, Φ the electric scalar potential, and ρ_V_ the density of volume charges. Under a fixed applied voltage at 50 kV, the distributions of electric field intensity **E** around the spherical electrode (radius of curvature 20 mm) with and without an artificial plasma channel are presented in Fig. 6. In the simulation, the artificial filament was regarded as a uniform plasma cylinder with a length of 6.75 cm (the radius of curvature r of the plasma channel was set at 0.05 mm and the electrical conductivity of the channel was assumed 100 siemens/s according to ref.^16^. When applying the high voltage of 50 kV to the spherical electrode only, the maximum electric field strength on the surface of the electrode in Fig. 6(a) is about 2 × 10^6^ V/m which is below the electric field threshold of (*E*_*r*_)_*sphere*_ = 4.5 × 10^6^ V/m for a streamer corona to occur, according to ref.^17^. This means there won’t be any streamer coronas with the spherical electrode alone. When the laser plasma channel was employed to couple with the high electric field in the current configuration (Fig. 6(b)), the resultant electric field along the artificial laser plasma channel is ~6.2 × 10^7^ V/m which is well above the threshold ((*E*_*r*_)_*laser*_ = 1.66 × 10^7^ V/m) for streamer coronas generation according to ref.^18^. Note that the calculated electric field threshold for streamer generation with laser plasma channel ((*E*_*r*_)_*laser*_) is higher than that of spherical electrode ((*E*_*r*_)_*sphere*_) under the same applied voltage. This may be due to the low conductivity of laser plasma channel as compared to metallic wire although laser plasma channel has sharp ends^15^. Strong electric field around the tip of plasma channel leads to discharges generation through avalanche ionization. Indeed, no streamer was observed in the absence of plasma channel in the experiment at 50 kV. But clear streamers were generated and observed along the plasma channel when the laser air plasma channel was added (Fig. 2(a)). These discharges are the sources of ionic wind. The numerical predication on the electric field distribution along laser plasma channel end (Figs 6 and 7) shows the electric field is large enough to generate corona discharges. As a consequence, clear wind flows were experimentally observed in Fig. 2(b–d). Therefore, the ionic wind in our experiment only originated from the laser guided discharges, not from the spherical electrode because of a low electric field from it. Indeed, no clear wind was observed from the spherical electrode.

In Fig. 6(b), there is weak field regime (near zero field) not far away from the exit of the electrode hole. When a large positive electric field is applied onto the electrode, the free electrons inside the plasma column will be pushed into the metallic electrode quickly leaving behind a channel of positive ions. The positive ions inside the plasma channel but outside the electrode will be pushed towards the tip of the channel. As a consequence, more and more positive ions will accumulate around the tip of the plasma channel. Such an accumulation will create a back electric field pointing towards the electrode along the plasma channel. At the same time, there is an electric field pointing outward along the plasma channel due to the positive charges inside the opening of the electrode. The two opposing fields will balance each other at some position resulting in a weak field regime as the simulation shows in Fig. 6(b). In reality, both temporal and spatial jitters of the pulses would smear out the near zero point because of its high repetition rate of 1 kHz. Even so, one can see this effect in the experiment; i.e. for a short distance outside the hole, there is no streamer at very high voltage (for example 50 kV in Fig. 2(a)) while after this short distance, streamers come out almost continuously along the plasma channel till its end tip. When the applied voltage is kept at 50 kV and the channel length is chosen from 40 mm to 70 mm, the simulated electric field distribution around the plasma channel end is shown in Fig. 7(a). The peak amplitude of electric field fluctuates almost at a constant value. The maximum electric field will generate maximum intensity of discharges leading to the maximum wind velocity being measured. The simulation agrees with the experimental observation in Fig. 4(a). By keeping the plasma channel length at 60 mm, the calculated peak electric field is linearly proportional to applied voltage as shown in Fig. 7(b) which can nicely explain the first linear region in Fig. 3. The air plasma channel^19–22^ created by femtosecond laser pulses through multiphoton/tunneling ionization can serve as a long conductor like a conducting wire offering early free electrons. When applying positive high voltage to the spherical copper electrode, the free electrons inside the external part of the plasma channel would be accelerated towards the electrode and positive ions are pushed towards the tip of the channel. In the early stage of increasing the applied voltage (<30 kV), the weak electric field at the tip of the filament resulted in a weak avalanche ionization for leader type of discharge generation, which may be responsible for the weak ionic wind generation in the first linear regime observed in Fig. 3 and simulated in Fig. 7(b). As the applied voltage keeps increasing, the positive ions at the sharp end of the plasma channel produce much higher positive electric field, as a consequence, significantly stronger leaders and streamers are generated leading to much stronger ionic wind generation at the high voltage in the second linear regime of Fig. 3. The simulation in Fig. 7(a) is based on the static field and plasma effect is not included. As shown in Fig. 7(c), the calculated electric field distribution in the direction perpendicular to the filament propagation predicts asymmetrical distribution of electric field at three positions of 1 mm, 5 mm and 10 mm before the plasma channel end. At the side (negative position value) where the electrode was connected to the power supply (shown in Figs 1 and 5(a)), the electric field is weaker. As a consequence, weaker discharges should be generated which agrees with the observation in Fig. 2(a). This may be the reason why an asymmetry of the ionic wind flows was observed (Figs 2(b–d) and 5(b)).

The ionic wind originates from the discharges from the laser air plasma channel (Fig. 5); hence, it can be generated at a distance (Fig. 4(a)) and optimized by shaping the laser induce plasma volume (Fig. 4(b)) and the external electric field^10^.

## Conclusion

We experimentally demonstrated that a strong ionic wind (>4 m/s) can be generated by coupling an external large electric field with a femtosecond laser-induced air plasma channel. The ionic wind relies upon the discharges along the plasma channel when an external high electric filed is applied. The dependence of the ionic wind velocity on the applied high voltages, plasma channel and laser characteristics have been systematically investigated. The experimental results are qualitatively understood with a numerical simulation. The approach reported in the work paves a way to optically generate ionic wind at a distance, even remotely.

## Electronic supplementary material

Supplementary video S1

Supplementary Information
