# Supplementary material for: Laser guided ionic wind
Source: Sci Rep. 2018 Sep 10;8:13511. doi: 10.1038/s41598-018-31993-3 (PMC6131152; doi:10.1038/s41598-018-31993-3)
Supplement: Supplementary file 2 — Supplementary Information [file 41598_2018_31993_MOESM2_ESM.docx]

**Laser guided ionic wind**

Shengzhe Du^1,§^, Tie-Jun Wang^1,*,§^, Zhongbin Zhu^1,§^, Yaoxiang Liu^1^, Na Chen^1^, Jianhao Zhang^1^, Hao Guo^1^, Haiyi Sun^1^, Jingjing Ju^1^, Cheng Wang^1^, Jiansheng Liu^1, #^ , See Leang Chin^2^, Ruxin Li^1, †^  and Zhizhan Xu^1,‡^

*^1^ State Key Laboratory of High Field Laser Physics, Shanghai Institute of Optics and Fine Mechanics, Chinese Academy of Sciences, China*

*^2^ Centre d’Optique, Photonique et Laser (COPL) and Département de physique, de génie physique et d’optique, Université Laval, Qu**ébec, Québec G1V 0A6, Canada*

[*tiejunwang@siom.ac.cn](mailto:*tiejunwang@siom.ac.cn),

^#^[michaeljs_liu@mail.siom.ac.cn](mailto:michaeljs_liu@mail.siom.ac.cn),

^†^[ruxinli@mail.shcnc.ac.cn](mailto:ruxinli@mail.shcnc.ac.cn)

^‡^zzxu@ mail.shcnc.ac.cn

^§^these authors contributed equally to the work

**Supplementary video: Movie S1**

Movement of laser guided ionic wind recorded a digital camera is shown in the supplementary video. The video was taken when laser plasma channel was created by focusing 6.8 mJ, 30 fs laser pulse with 40 cm focal length lens and DC high voltage of 20 kV was applied onto the channel through the designed electrode.
